# Decreased Kinase Activity of the VEGFR3 Variant c.3175G>C Associated with Primary Lymphedema

**DOI:** 10.3390/cimb48010068

**Published:** 2026-01-08

**Authors:** Yuliya V. Filina, Maria A. Zolotykh, Regina R. Miftakhova

**Affiliations:** Institute of Fundamental Medicine and Biology, Kazan (Volga Region) Federal University, Kazan 420008, Russia; mazolotykh@kpfu.ru (M.A.Z.); rermiftahova@kpfu.ru (R.R.M.)

**Keywords:** VEGFR3, tyrosine kinase, lymphedema

## Abstract

Vascular endothelial growth factor receptor 3 (VEGFR3) assumes a pivotal role in regulating the development and maintaining the structural integrity of the lymphatic system. Decreased activity of VEGFR3 can precipitate aplasia or hypoplasia of lymphatic system components, culminating in primary lymphedema. To date, numerous genetic variants have been identified within the *FLT4* gene, which encodes VEGFR3; however, the majority of these remain uncharacterised and are classified as ‘variants of uncertain significance’. In preceding investigations involving *FLT4* sequence analysis conducted on individuals presenting with primary lymphedema, we identified several rare genetic variants that possess the potential to modulate the functional activity of VEGFR3, including the heterozygous variant c.3175G>C (p.A1059P). Preliminary assessments encompassing clinical characteristics, family history, and predictive computational algorithms indicated that this variant was likely pathogenic. Consequently, this study presents the results of functional evaluation of the mutant VEGFR3 activity in cell models overexpressing the *FLT4* variant c.3175G>C. VEGFC-dependent VEGFR3 phosphorylation and *FLT4* expression were reduced in cells with c.3175G>C *FLT4* variant compared to wild-type, confirming the pathogenic role of c.3175G>C in primary lymphedema.

## 1. Introduction

Primary lymphedema is a rare, predominantly hereditary condition with an incidence of 1 in 6000 to 10,000 individuals. It is characterized by progressive edema with phenotypic and genetic heterogeneity. Congenital lymphedema is marked by edema that is present at birth or develops shortly thereafter, occurring in approximately 1 in 6000 children [1]. Recent studies of families with hereditary forms of lymphedema have identified 13 genes associated with the development of primary lymphedema: *FLT4* (*VEGFR3*), *GJC2*, *FOXC2*, *SOX18*, *GATA2*, *CCBE1*, *PTPN14*, *KIF1*, *VEGFC*, *HGF*, *MET*, *PIEZO1*, and *EPHB4* [2,3,4,5]. It is important to note that most of the proteins encoded by these genes are involved in the VEGFC/VEGFR3 signaling pathway [6].

Vascular endothelial growth factor receptor 3 (VEGFR3) is a critical regulator of the development of the lymphatic and circulatory systems during embryogenesis, as well as the maintenance of lymphatic vessel structure throughout life [7,8]. The VEGFR3-encoding gene, *FLT4* (NM_182925.5), consists of 30 exons and encodes a 1363-amino acid protein that contains seven immunoglobulin domains, a transmembrane domain, and tyrosine kinase domains, serving as a receptor for Vascular endothelial growth factors C and D (VEGFC and VEGFD) in human [9,10,11]. Pathogenic alterations in *FLT4* are linked to cardiovascular disorders in humans; specifically, truncation and frameshift mutations in the immunoglobulin domains are associated with congenital heart defects [6], while pathogenic mutations in the tyrosine kinase domain are linked to primary lymphedema type 1A (Milroy disease) [12].

A key event in the signal transduction within this molecular pathway is the interaction between the VEGFR3 receptor and the VEGFC ligand. Stimulation by VEGFC leads to the phosphorylation of the receptor and subsequent activation of numerous signaling pathways that influence the proliferation and migration of lymphatic endothelial cells. Pathogenic mutations in the tyrosine kinase domain of VEGFR3 can impair ATP binding or inhibit receptor phosphorylation, thereby preventing downstream signaling and disrupting the development of the lymphatic system [6]. Thus, the normal structure of the activation loop, along with the substrate and ATP binding sites in the tyrosine kinase domain, is essential for effective intracellular signal transduction.

Despite the well-understood mechanism underlying the disease’s development, assessing the clinical significance of individual genetic variants remains a challenging task. In previous analyses of the *FLT4* sequence in patients with primary lymphedema, we identified several rare genetic variants that may affect the functional activity of VEGFR3, including the heterozygous variant c.3175G>C (p.A1059P) [13]. An initial evaluation of the clinical features of these patients, family history, and data from predictive algorithms suggested that the identified variants are likely pathogenic. The objective of this study was to assess the functional activity of VEGFR3 in model cells that overexpress the mutant *FLT4* variant c.3175G>C to confidently confirm the pathogenicity of this variant.

## 2. Materials and Methods

### 2.1. Plasmid Preparation

The pHAGE-FLT4 plasmid (http://n2t.net/addgene:116745 (accessed on 29 November 2023); RRID:Addgene_116745 [14]), which encodes the wild-type *FLT4* gene sequence, was employed for transgene production. Site-directed mutagenesis (SDM) utilizing QuikChange Site-Directed Mutagenesis Kit (Agilent, Santa Clara, CA, USA) was implemented to generate a mutant variant of *FLT4* featuring a G>C substitution at position 6622 of pHAGE-FLT4 (corresponding to *FLT4* c.3175G>C). The mutant sequence was generated following the manufacturer’s protocol.

Plasmids were amplified in *Escherichia coli* XL10-gold cells, as supplied with the QuikChange Site-Directed Mutagenesis Kit, in accordance with the manufacturer’s instructions. Plasmid DNA was extracted using the Plasmid Midiprep 2.0 Kit (Evrogen, Moscow, Russia), precipitated with ethanol, and stored at −20 °C for subsequent lentivirus production. The nucleotide substitution was confirmed by Sanger sequencing of the target regions.

### 2.2. Primer Design

Primers for the SDM reaction were designed using the QuickChange Primer Design tool (Agilent, USA). Additionally, primers for Sanger sequencing and quantitative PCR (qPCR) were designed utilizing PrimerQuest tool (https://www.idtdna.com/pages/tools/primerquest (accessed on 19 December 2023), IDT DNA, Coralville, IA, USA). All primers were synthesized by Genterra JSC (Moscow, Russia). The primer sequences are provided in Table 1.

### 2.3. Lentivirus Production

Lentiviral particles were produced through transient transfection of HEK293FT cells using the calcium phosphate method [15]. The packaging plasmid psPAX2 was generously provided by Didier Trono (http://n2t.net/addgene:12260 (accessed on 29 November 2023); RRID:Addgene_12260), and the envelope plasmid pCMV-VSV-G was a gift from Bob Weinberg (http://n2t.net/addgene:8454 (accessed on 29 November 2023); RRID:Addgene_8454 [16]).

Lentiviruses were concentrated by ultracentrifugation on an Optima L-90K (Beckman Coulter, Brea, CA, USA) at 120,000× *g* for 2 h and subsequently stored at −86 °C. The lentiviral titer was measured by fluorescence-activated cell sorting (FACS) using serial dilutions on HEK293FT cells. In the pHAGE-FLT4 vector, the *FLT4* and *EGFP* genes are separated by an internal ribosome entry site (IRES) element and are under the control of the EF1A promoter, which facilitates their co-transcription. Thus, lentivirus titer, preliminary transduction efficiency estimation, and cell sorting were based on GFP fluorescence.

### 2.4. Cell Model

Cell lines overexpressing *FLT4* were generated through the transduction of PC-3 cells with lentiviral vectors encoding either wild-type *FLT4* (denoted as WT in subsequent text) or the mutant *FLT4* variant c.3175G>C (designated as c.3175G>C in subsequent text). For transduction, PC-3 cells were seeded in 24-well plates, and after 16 h, 10 μg/mL protamine sulfate along with 10^6^ transducing units (TU) of lentivirus were added per 200,000 cells.

Transduction efficiency was analyzed by flow cytometry 48 h post-transduction (designated as passage 1, p1). At passages 6 and 10, cells were sorted using FACS Aria III to target GFP-positive cells with a purity of no less than 90%. The final number of integrated lentivirus copies per cell was determined using the TransLv Lentivirus qPCR Titration Kit (TransGen Biotech, Beijing, China).

Cells were cultured in RPMI-1640 medium supplemented with 2 mM L-glutamine and a penicillin-streptomycin mixture (all from Paneco, Moscow, Russia), and 10% fetal bovine serum (Biosera, Cholet, France) under standard conditions. Non-transduced PC-3 cells served as the control (denoted as CTRL/control in subsequent text).

### 2.5. VEGFR3 Pathway Activation

To analyze short-term VEGFR3 pathway activation, sixteen to eighteen hours prior to stimulation, cell culture media were replaced with RPMI-1640 containing L-glutamine and penicillin-streptomycin without fetal bovine serum. Cells were incubated with 100 ng/mL VEGFC (Wuhan Fine Biotech, Wuhan, China) for durations of 10, 20, or 30 min at 37 °C. Following this incubation, cells were washed with phosphate-buffered saline devoid of Ca^2+^ and Mg^2+^, treated with VEGFC, transferred to ice, and lysed using cold RIPA buffer supplemented with protease inhibitor cocktail. Non-treated cells served as the control (denoted as 0 s in subsequent text and figures).

Samples underwent separation via 10% PAGE under denaturing conditions before being transferred to a PVDF membrane and probed with antibodies specific to phosphorylated VEGFR3 (Tyr1230, #AF3676, Affinity Biosciences, Liyang, China), phosphorylated AKT (Ser273, #4060, Cell Signaling Technologies, Danvers, MA, USA), total VEGFR3 (#AF4201, Affinity Biosciences, China), total AKT (#AF6259, Affinity Biosciences, China), and GAPDH (#CAB932Hu01, Cloud-clone, Wuhan, China) as a loading control.

### 2.6. Gene and Protein Expression Analysis

Gene expression was assessed through qPCR to evaluate the long-term effects of VEGFC. Total RNA was extracted from 1–2 × 10^6^ PC-3 cells that had been incubated in RPMI-1640 medium supplemented with 2 mM L-glutamine, penicillin-streptomycin mixture, 2% FBS and 1000 ng/mL of VEGFC for 72 h in standard culture conditions. Complementary DNA was synthesized using the RevertAid RT Kit (Thermo Fisher Scientific, Waltham, MA, USA) from 400 to 500 ng of total RNA per reaction. The reaction mixture (Evrogen, Moscow, Russia) included the intercalating dye SYBR Green for the qPCR analysis. The results were analyzed using ACTB expression as a reference and the ΔΔCt method in CFX Manager Software version 3.1 (Bio-Rad Laboratories, Hercules, CA, USA).

Protein expression was assessed through Western blot analysis after 72 h of incubation with 1000 ng/mL of VEGFC as described above in Section 2.5.

### 2.7. Statistical Analysis

Statistical analysis was conducted using GraphPad Prism 8.0 (GraphPad Software, USA). One-way ANOVA with Tukey’s multiple comparisons test or two-way ANOVA with the two-stage linear step-up procedure of Benjamini, Krieger, and Yekutieli was employed as appropriate. Data are presented as mean ± SEM, with *p*-values indicated as follows: ns—*p* > 0.05, * *p* ≤ 0.05, ** *p* ≤ 0.01, *** *p* ≤ 0.005, and **** *p* ≤ 0.001.

## 3. Results

### 3.1. Transgene Production and Model Cells Generation

**Figure 1:**
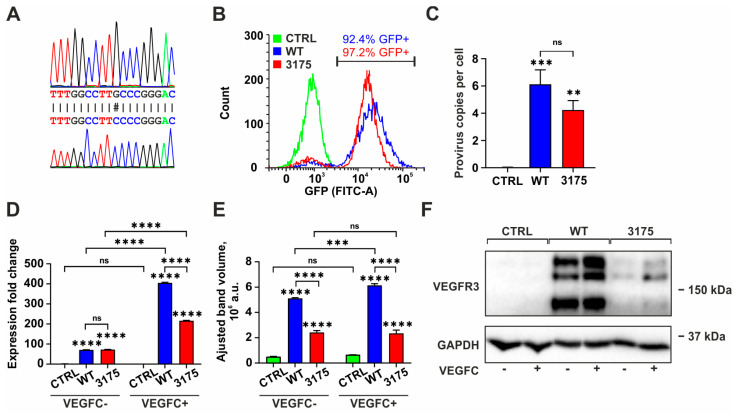
Transgene and Cell Model Production and Analysis: Results of the Sanger sequencing of the initial pHAGE-FLT4 plasmid (top) and the pHAGE-FLT4 plasmid containing the *FLT4* c.3175G>C variant, obtained through site-directed mutagenesis (SDM) (bottom); # denotes the mismatched nucleotide (**A**); representative results of flow cytometry analysis (FACS) of PC-3 cells transduced with lentiviruses carrying wild-type FLT4 (WT) and the FLT4 variant c.3175G>C (3175), following cell sorting (**B**); proviral copy number in PC-3 cells before (CTRL) and after transduction with lentiviruses carrying wild-type FLT4 (WT) and the FLT4 variant c.3175G>C (3175), as well as after two rounds of cell sorting ((**C**), *n* = 3, ** *p* ≤ 0.01, *** *p* ≤ 0.005 in comparison to CTRL; differences between WT and 3175 are non-significant (ns); one-way ANOVA and Tukey’s multiple comparisons test); expression of the FLT4 gene (**D**) and VEGFR3 protein ((**E**)—results of densitometry analysis; (**F**)—representative Western blot image) in WT and c.3175G>C (3175) cells, incubated with VEGFC (VEGFC+) or without VEGFC (VEGFC−) for 72 h (D: *n* = 3, E: *n* = 2, *** *p* ≤ 0.005 and **** *p* ≤ 0.001; two-way ANOVA with the two-stage linear step-up procedure of Benjamini, Krieger, and Yekutieli).

Site-directed mutagenesis was employed to generate pHAGE-FLT4 plasmids containing substitutions corresponding to the *FLT4* variant c.3175G>C. The nucleotide sequence of the region encoding the tyrosine kinase domain was verified through Sanger sequencing (Figure 1A). Both the resultant plasmid and the original pHAGE-FLT4 were utilized to assemble lentiviruses for the transduction of PC-3 cells.

The lentivirus titer, measured in HEK293FT cells, was found to be 1.3 × 10^8^ ± 6.7 × 10^7^ TU/mL for the wild type (WT) and 1.7 × 10^8^ ± 8.1 × 10^7^ TU/mL for the c.3175G>C variant (*n* = 2; data not presented). Initially, 200,000 PC-3 cells were transduced with 10^6^ TU of lentivirus to achieve a multiplicity of infection of 5.

At 72 h post-transduction, the proportion of GFP-positive cells for WT and c.3175G>C cells was 25.7 ± 1.1% and 12.0 ± 1.2%, respectively (*n* = 3). GFP-positive cells underwent two rounds of sorting to achieve a target purity of no less than 90% (Figure 1B). The provirus copy number was assessed in cells following two rounds of sorting at passages 14, 20, and 37. The results were as follows: 0.03 ± 0.03 for the control, 6.12 ± 1.07 for WT, and 4.24 ± 0.69 for the c.3175G>C variant (Figure 1C). Consequently, stable cell lines expressing both the wild-type and mutant *FLT4* variant c.3175G>C were established for subsequent analysis.

### 3.2. VEGFR3 Activation

#### 3.2.1. Challenging Effect of VEGFC on the VEGFR3 Pathway Activity and FLT4 Expression Is Reduced in Cells with C.3175G>C FLT4 Variant

The primary natural ligand of VEGFR3, VEGFC, was utilized to assess the activation of the VEGFR3 pathway. To analyze the long-term stimulation effects, model cells were incubated with VEGFC for 72 h, after which lysates were examined using qPCR and Western blot analysis.

The results from qPCR indicated that *FLT4* expression in both variants of transduced cells was approximately 70 times higher than that observed in control PC-3 cells (Figure 1D). Furthermore, incubation with VEGFC further enhanced *FLT4* expression: from 70.8 ± 1.0 to 404.7 ± 3.4 in wild-type cells and from 72.5 ± 1.0 to 216.0 ± 1.9 in cells with the c.3175G>C variant. However, the level of *FLT4* expression in VEGFC-treated mutant cells was significantly lower than that in VEGFC-treated WT cells.

Western blot analysis (Figure 1E,F) corroborated these findings, revealing two notable distinctions: first, the expression of VEGFR3 at the protein level in c.3175G>C cells was significantly lower than in WT cells; second, treatment with VEGFC for 72 h did not result in a significant increase in the total VEGFR3 level in c.3175G>C cells.

#### 3.2.2. FLT4 c.3175G>C Unable to Rapidly Activate Under VEGFC Treatment

**Figure 2:**
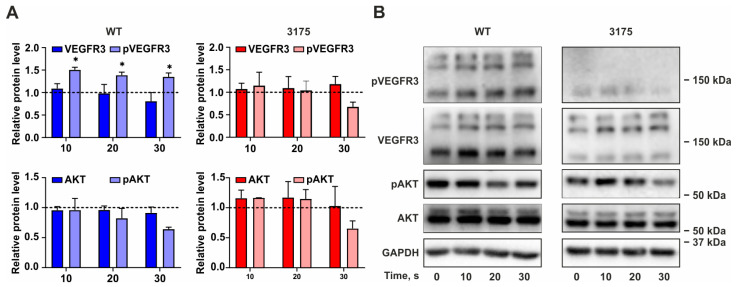
VEGFC-Dependent Short-Term Activation of the VEGFR3 Pathway in WT and c.3175G>C (3175) Cells: Densitometry analysis of Western blot results (**A**) and representative Western blot images (**B**); relative protein levels are calculated as the ratio of the protein level at the indicated time point to the level of the protein at time point 0 in the corresponding experiment, taken as 1 (dashed line); ((**A**)—*n* = 3, * *p* ≤ 0.05; two-way ANOVA with the two-stage linear step-up procedure of Benjamini, Krieger, and Yekutieli).

To investigate the short-term effects of VEGFC on the activation of the VEGFR3 pathway, WT and c.3175G>C cells were incubated with VEGFC for 10–30 min, and the levels of phospho-VEGFR3 and phospho-panAKT were analyzed in cell lysates via Western blot (Figure 2). In WT cells, the level of phosphorylated VEGFR3 increased at the 10 min mark following VEGFC treatment and remained stable at both the 20 min and 30 min intervals. In contrast, only a very low level of phospho-VEGFR3 was detected in c.3175G>C cells throughout the entire observation period, from 0 to 30 min.

Additionally, we assessed AKT activation levels; however, no significant effects were documented under short-term VEGFC treatment in both cell lines.

## 4. Discussion

The tyrosine kinase domain of vascular endothelial growth factor receptor 3 (VEGFR3), encompassing amino acids 845 to 1173 as indicated in the UniProt entry P35916, exhibits a high degree of conservation across species and within the receptor tyrosine kinase family [17,18]. The integrity of the activation loop, as well as the substrate and ATP binding sites, is essential for effective intracellular signal transduction. The alanine residue at position 1059 is located within the activation loop; thus, missense mutations at this site could directly impact kinase activity. Non-conservative amino acid substitutions, such as alanine to proline [19], are likely to alter the conformation of the active site. Most in silico algorithms that incorporate evolutionary conservation analyses [20] predict a damaging or deleterious effect associated with this substitution.

The variant c.3175C>G (p.A1059P) has been previously documented by Liu and Gao [21] as well as in our own research [13], and it has been classified as “likely pathogenic.” In both instances, this variant was identified in patients with isolated Milroy disease, who presented with edema in the lower limbs below the knee.

Despite this evidence, it remains impossible to accurately assess the pathogenicity of the variant without functional tests. This study employs model cell lines that overexpress either wild-type *FLT4* or the mutant *FLT4* variant c.3175G>C to evaluate its functional activity, thereby elucidating the pathogenic role of c.3175G>C in VEGFR3 dysfunction and the development of primary lymphedema.

In our investigation, we analyzed the effects of VEGFC on VEGFR3 activity and expression. VEGFC serves as the primary ligand that activates VEGFR3 in lymphatic endothelial cells (LECs). It is crucial for lymphatic development, and the VEGFR3/VEGFC signaling axis plays a particularly significant role during lymphatic growth when LECs begin to sprout from the cardinal vein [22].

A range of features associated with the c.3175G>C variant was evaluated in comparison to wild-type *FLT4*. Initially, in cells expressing c.3175G>C, the stimulatory effect of VEGFC on *FLT4* gene expression and VEGFR3 protein expression was diminished (Figure 2). It has been demonstrated that VEGFC/VEGFR3 signaling enhances VEGFR3 expression through a mechanism that is not yet fully understood [23]. qPCR analysis revealed that this mechanism was partially disrupted, resulting in reduced stimulation of *FLT4* expression by VEGFC after 72 h of treatment.

A similar trend was observed at the protein level; however, in this experiment, receptor levels did not change in response to ligand treatment. This observation may be attributed to translation or post-translational modifications of the receptor. Currently, there is limited information regarding disrupted VEGFR3 translation due to a single nucleotide change, indicating that further investigation is warranted. Previous studies have discussed post-translational modifications in relation to pathogenic variants of *FLT4*. Three forms of VEGFR3 have been identified in human cells: a 195 kDa glycosylated full-length VEGFR3, a 175 kDa partially glycosylated protein, and a 125 kDa product resulting from proteolytic cleavage during receptor maturation. Karkkainen et al. demonstrated that the predominant form of wild-type VEGFR3 is the 125 kDa protein, whereas the 175 kDa peptide is more abundant in P1114L and L1044P mutants [24]. We observed similar properties: for the wild-type, the major band corresponded to about 125 kDa protein, whereas in the c.3175G>C, variant the bulk of the protein is represented by 175 kDa form (Figure 1F and Figure 2B).

Receptor tyrosine kinases, including VEGFR3, are activated through ligand-induced dimerization, which brings together the cytoplasmic tyrosine kinase domains [25]. This process stimulates trans-autophosphorylation of tyrosine residues within the kinase activation loop and perimembrane region, leading to conformational changes that stabilize the active state. Consequently, autophosphorylation represents a critical event in intracellular receptor tyrosine kinase signaling. We examined the short-term effects of VEGFC on phosphorylation of the c.3175G>C variant and found this phosphorylation to be reduced as well. Prior studies utilizing model cell lines have indicated activation of wild-type VEGFR3 within 5–10 min of VEGFC stimulation [24,26]. In wild-type cells, we observed stable phosphorylation of VEGFR3 during 10-, 20-, and 30 min treatments with VEGFC. In contrast, in cells expressing the c.3175G>C variant, the level of phosphorylated VEGFR3 was low and did not change compared to non-activated cells (point 0, Figure 2).

Phosphotyrosines recruit signaling molecules containing SH2 and PTB domains, which leads to the activation of the Ras, PI3K, and PLCγ signaling pathways [25,27]. The serine/threonine kinase AKT is a key signal transducer in the PI3K pathway, promoting cell survival and angiogenesis. To further investigate intracellular signal transduction from the VEGFC/VEGFR3 interaction, we analyzed AKT phosphorylation. Our findings indicated that the level of phosphorylated AKT did not change over the studied time interval in either wild-type or c.3175G>C cells.

## 5. Conclusions

Numerous genetic variants have been identified in patients with Milroy disease, the majority of which are classified as variants of uncertain significance. The pathogenic role of novel genetic variants can generally be confirmed through a combination of several approaches: population analysis, family segregation analysis, functional and laboratory tests, and mathematical modeling [28]. In cases of lymphedema and other similar rare diseases, population analysis is often impractical; mutations in well-studied genes typically occur in individual patients, making it challenging to gather a sufficient sample size for statistical calculations. Family segregation analysis is further complicated by incomplete penetrance and the presence of sporadic cases of the disease, while mathematical algorithms frequently yield conflicting results. Consequently, functional analyses utilizing model systems provide the most reliable insights into protein dysfunction that may lead to disease development.

However, such studies have limitations. First, when employing tumor-derived cells, such as PC-3, one may observe an elevated overall activity of components within the receptor tyrosine kinase signaling pathway. In this study, we utilized PC-3-derived cell lines that overexpress either the wild-type or the mutant variants of *FLT4* to demonstrate that the c.3175G>C *FLT4* variant exhibited reduced activity, supporting its association with the development of primary lymphedema. We hypothesize that the activation of the mutant variant may also be delayed; thus, longer time intervals could be examined to elucidate the precise kinetic parameters of the tyrosine kinase. The partial preservation of the stimulatory effect of VEGFC on *FLT4* expression further supports this hypothesis and may help explain the incomplete penetrance associated with the c.3175G>C variant.

## Figures and Tables

**Table 1:** Primer sequences.

No	Method	Target	Direction	Sequence 5′-3′ ^1^
1	SDM	pHAGE-FLT4	Forward	GATCTGTGACTTTGGCCTT**C**CCCGGGACAT
			Reverse	ATGTCCCGGG**G**AAGGCCAAAGTCACAGATC
2	Sequencing	pHAGE-FLT4 (region 6238-6735)	Forward	AAGTACGGCAACCTCTCC
			Reverse	GTCACTCTGCGTGGTGTA
3	qPCR	*FLT4* (NM_182925.5)	Forward	GGACTCCTGGACGGCCT
			Reverse	GGTGTCGATGACGTGTGACT
4		*ACTB* (NM_001101.5)	Forward	GCCCTGAGGCACTCTTCCA
			Reverse	CGGATGTCCACGTCACACTTCA

^1^ in SDM primers mismatched nucleotides are highlighted in bold.

## Data Availability

The original contributions presented in this study are included in the article. Further inquiries can be directed to the corresponding author(s).
